# High-Precision Positioning and Rotation Angle Estimation for a Target Pallet Based on BeiDou Navigation Satellite System and Vision

**DOI:** 10.3390/s24165330

**Published:** 2024-08-17

**Authors:** Deqiang Meng, Yufei Ren, Xinli Yu, Xiaoxv Yin, Wenming Wang, Junhui Men

**Affiliations:** Qilu Aerospace Information Research Institute, Jinan 250100, China; mengdq@aircas.ac.cn (D.M.); yuxinli@aircas.ac.cn (X.Y.); yinxiaoxu2017@163.com (X.Y.); wwm_96@163.com (W.W.); menjh@aircas.ac.cn (J.M.)

**Keywords:** unmanned forklift, automated unloading, target pallet detection and localization, rotation angle estimation, BDS and vision, deep learning

## Abstract

In outdoor unmanned forklift unloading scenarios, pallet detection and localization face challenges posed by uncontrollable lighting conditions. Furthermore, the stacking and close arrangement of pallets also increase the difficulty of positioning a target pallet. To solve these problems, a method for high-precision positioning and rotation angle estimation for a target pallet using the BeiDou Navigation Satellite System (BDS) and vision is proposed. Deep dual-resolution networks (DDRNets) are used to segment the pallet from depth images and RGB images. Then, keypoints for calculating the position and rotation angle are extracted and further combined with the 3D point cloud data to achieve accurate pallet positioning. Constraining the pixel coordinates and depth coordinates of the center point of the pallet and setting the priority of the pallet according to the unloading direction allow the target pallet to be identified from multiple pallets. The positioning of the target pallet in the forklift navigation coordinate system is achieved by integrating BDS positioning data through coordinate transformation. This method is robust in response to lighting influences and can accurately locate the target pallet. The experimental results show that the pallet positioning error is less than 20 mm, and the rotation angle error is less than 0.37°, which meets the accuracy requirements for automated forklift operations.

## 1. Introduction

With the rapid development of modern logistics, automated handling equipment, especially unmanned forklifts, has been widely used, which can improve the efficiency of cargo unloading and reduce operating costs [1,2,3]. As one of the key technologies for autonomous operation of forklifts, pallet detection and localization have garnered increasing attention [4]. In outdoor unmanned unloading scenarios, the position of a pallet is highly uncertain and is influenced by uncontrollable lighting conditions, making accurate detection and localization of the pallet more challenging.

Researchers have carried out a lot of research work for pallet detection and localization. The mainstream methods can be categorized into three types: vision-based, point-cloud-based, or a combination of both. Early vision-based detection methods were based on the natural features of the pallet to design segmentation strategies to segment the pallet from the image background and combined with geometric computation methods to realize pallet localization [5,6,7]. However, these methods are susceptible to background interference, and their robustness in complex logistics scenarios is not ideal [8]. Another approach involves adding reference markers to the surface of the pallet to enhance its recognition features, thereby achieving more precise pallet identification and localization [9,10]. However, this method is susceptible to marker deterioration and is not suitable for logistics scenarios with high pallet circulation. In recent years, object detection algorithms based on deep learning have been used to detect pallets in 2D images. Compared with early detection methods based on traditional machine vision, they have improved the speed and accuracy of pallet detection, providing an efficient solution for pallet detection in indoor environments [11,12,13,14].

Point-cloud-based methods use plan segmentation to process the point cloud data, and then use template matching or point cloud registration to achieve pallet localization [15,16,17,18]. Compared with vision-based methods, the point-cloud-based methods have the advantage of resisting the effect of lighting. However, processing point cloud data usually requires a large amount of computing resources, and the quality of the point cloud data directly affects the accuracy of pallet localization, which puts more stringent requirements on the performance of sensors and computing units.

The methods based on vision and point clouds combine the advantages of both and can not only use deep learning algorithms to accurately identify pallets in 2D images, but also use point cloud processing methods to achieve precise positioning of pallets in 3D space [19,20,21,22]. These methods reduce the need for computational resources and achieve a balance between speed and accuracy. However, these methods rely on extracting the pallet from RGB images. When the camera cannot stably obtain valid RGB images due to the influence of uncontrollable outdoor lighting conditions, these methods become ineffective, making them unsuitable for outdoor scenarios.

Furthermore, most research focuses on the identification and positioning of single pallets [23], while ignoring the situation of stacked and closely arranged pallets in actual unloading scenarios. There is a lack of research on target pallet detection and localization.

To solve these problems, a method for high-precision positioning and rotation angle estimation for a target pallet using the BeiDou Navigation Satellite System (BDS) and vision is proposed. The main contributions of this method are as follows: (1) Deep dual-resolution networks (DDRNets) are used to segment the pallet in 2D images. Then the keypoints for calculating the position and rotation angle are extracted and further combined with the 3D point cloud data to achieve pallet positioning. This method takes into account the speed and accuracy of pallet positioning and meets the real-time requirements of unmanned forklifts. (2) The method achieves accurate positioning of the target pallet in the case of stacked and closely arranged pallets by constraining the pixel coordinates and depth coordinates of the center point of the pallet and setting the priority of the pallet according to the unloading direction. (3) This method uses depth images with strong light resistance as the processing object and can effectively cope with the impact of lighting changes. Moreover, this method can work normally without lighting conditions and is suitable for autonomous forklift operations in outdoor scenes.

## 2. Method

**Figure 1:**
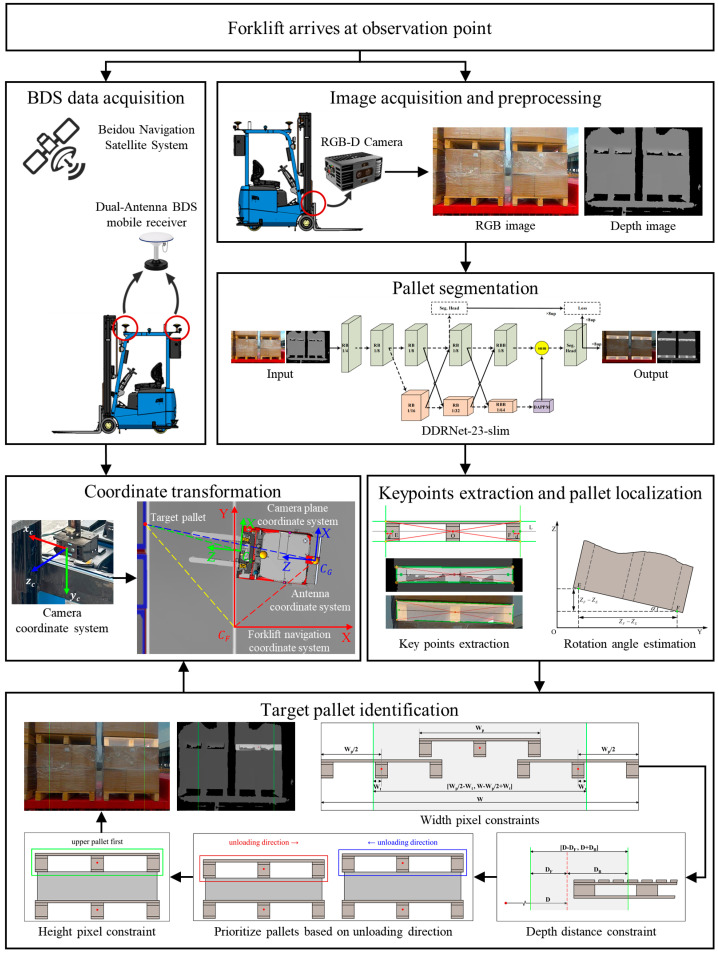
Flowchart of the overall methodology.

The flowchart of the proposed method in this article is shown in Figure 1. It consists of six phases: image acquisition and preprocessing, pallet segmentation, keypoint extraction and pallet localization, target pallet identification, BDS data acquisition, and coordinate transformation. Firstly, RGB images and depth images of the pallet are captured using an RGB-D camera at the forklift observation location. The Deep Dual-resolution Network-23-slim (DDRNet-23-slim) [24] is used to segment the pallet from 2D images. Then the pallet boundary straight lines are extracted using Progressive Probabilistic Hough Transform (PPHT) [25], and the pallet endpoints are obtained by clustering the intersections of these lines using the K-means clustering algorithm [26]. The intersection of the two diagonals and the correction point of the boundary center point on both sides are extracted as keypoints. Subsequently, the position and rotation angle of the pallet are calculated based on point cloud data indexing by keypoints. Constraining the pixel coordinates and depth coordinates of the center point of the pallet and setting the priority of the pallet according to the unloading direction allow the target pallet to be identified from many pallets. The position and yaw angle of the forklift are acquired by the dual-antenna BDS mobile receiver, and finally, the positioning of the target pallet in the forklift navigation coordinate system is achieved through coordinate transformation [27,28].

### 2.1. Image Acquisition and Preprocessing

**Figure 2:**
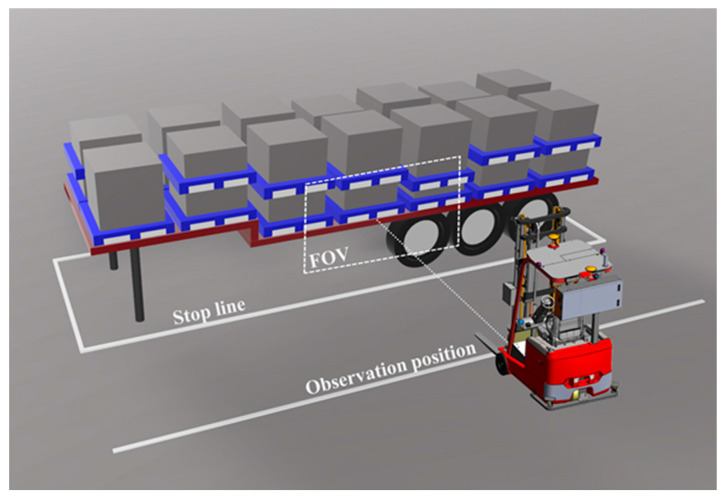
Schematic diagram of observation position.

After the flatbed truck stops along the stop line on the ground, the unmanned forklift receives the unloading task and arrives at the observation position on the side of the flatbed truck. Each pallet corresponds to an approximate observation position, which is set in advance based on the stop line. The observation position is shown in Figure 2, and D denotes the vertical distance between the observation position and the stop line. Affected by the camera’s field of view (FOV), ranging accuracy, and fork arm length, the value of D must meet the following conditions: (1) Even if the flatbed truck is parked deviated, the pallet must appear within the camera’s field of view. (2) The camera ranging accuracy decreases as the observation distance increases, so the observation position should not be too far from the stop line. (3) The observation position should not be too close to the stop line to avoid contact between the fork arm and the flatbed truck, which may lead to safety accidents.

**Figure 3:**
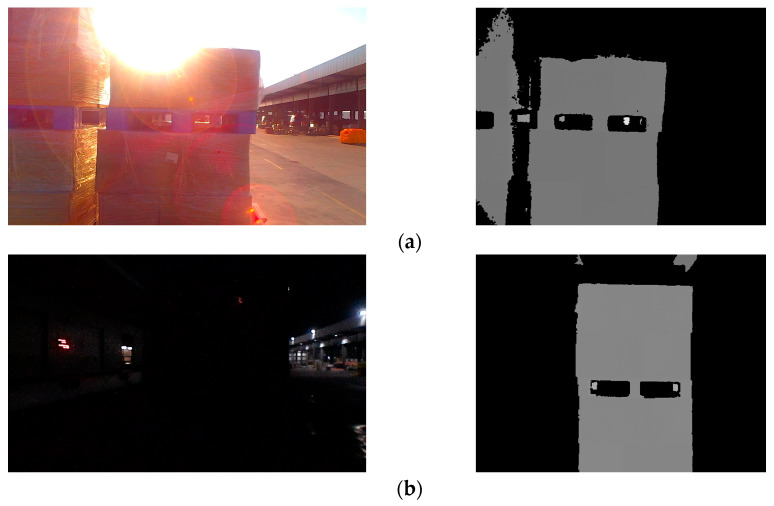

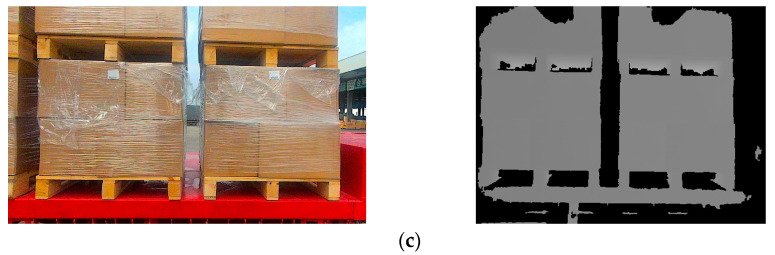
RGB images and depth images of pallets under different lighting conditions. (**a**) Pallets under strong light condition; (**b**) pallets under no light condition; (**c**) pallets under good light condition.

The unmanned forklift is equipped with an RGB-D camera to capture RGB images and depth images of the pallet. The depth channel of RGB-D camera has a field of view similar to that of the color channel and is not affected by variant illumination. Even in dark environments, the RGB-D camera can stably acquire depth images. Figure 3 illustrates the RGB image and depth image provided by an RGB-D camera. In order to reduce the interference pixels of other objects in the depth image, the depth threshold $D_{T}$ is set with D as the reference. By eliminating depth data larger than $D_{T}$, most non-target-object pixels are filtered out. In addition, for the purpose of reducing the interference caused by the jitter of the depth data at the edge of the pallet, Gaussian filtering on a large scale is used to smooth the depth image and reduce the noise.

### 2.2. Pallet Segmentation

There are many types of pallets used in the logistics industry. The circulation of pallets among warehouses is very strong, which leads to varying degrees of wear and deformation of pallets. Additionally, the pallets are tilted at small angles or partially obscured by plastic film and are affected by different conditions. It is difficult for traditional image processing methods to cope with these situations. On the contrary, image segmentation methods based on deep learning extract features through multi-layer neural networks, which can achieve automated target classification and are more suitable for the above situations.

For unmanned forklifts, it is not desirable to spend a lot of inference time to obtain segmentation results with high precision. Therefore, we apply the lightweight DDRNet-23-slim semantic segmentation model to the pallet segmentation. DDRNet-23-slim features deep dual-resolution networks as efficient backbones for real-time segmentation, providing a trade-off between accuracy and efficiency by scaling model width and depth [24]. Table 1 shows the architecture of DDRNet-23-slim.

Plastic pallets and wooden pallets are widely used in the logistics industry. Table 2 shows the specific information about the pallets used in this article.

**Figure 4:**
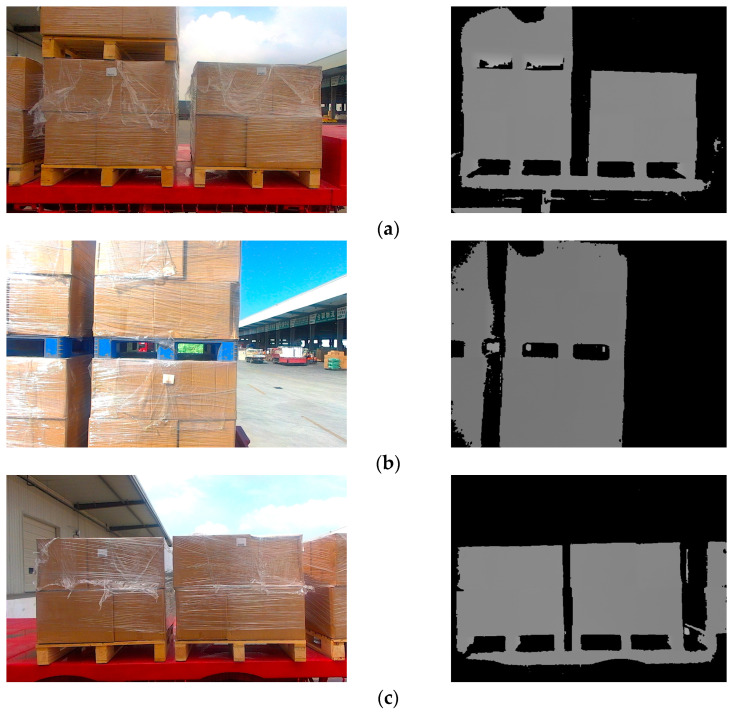
RGB images and depth images of pallets under different conditions. (**a**) Pallets at different heights; (**b**) pallets partially covered by plastic film; (**c**) pallets at small tilt angles.

In order to match the real environment, pallets under different conditions were collected in the dataset. In addition to pallets under different lighting conditions, pallets partially covered by plastic film, at different heights, or at small tilt angles were also collected, as shown in Figure 4. 

**Figure 5:**
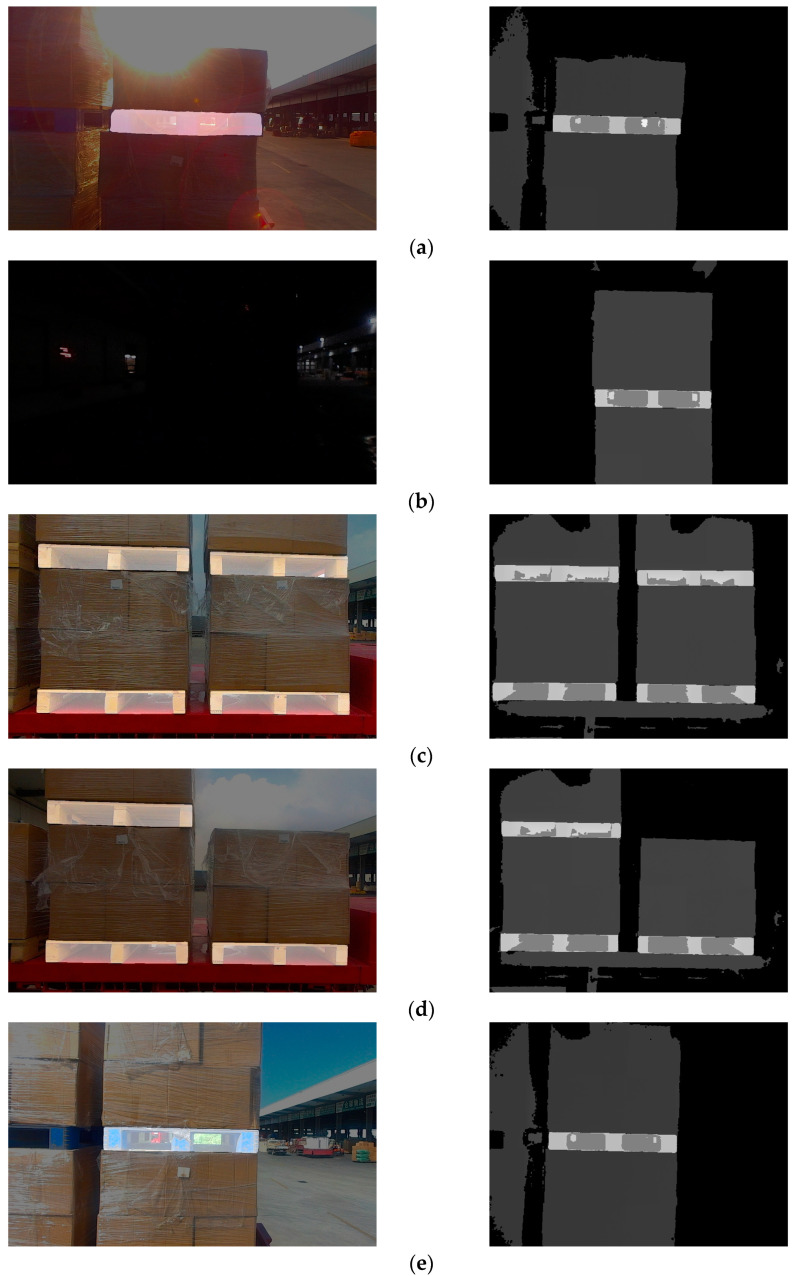

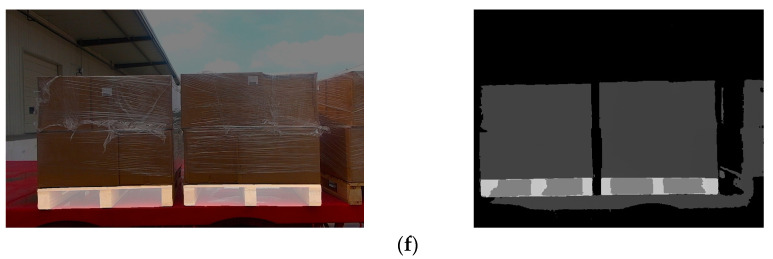
Segmentation results of pallets under different conditions based on RGB and depth images. (**a**) Pallets under strong light condition; (**b**) pallets under no light condition; (**c**) pallets under good light condition; (**d**) pallets at different heights; (**e**) pallets partially covered by plastic film; (**f**) pallets at small tilt angles.

The segmentation results are shown in Figure 5.

### 2.3. Keypoint Extraction and Pallet Localization

**Figure 6:**
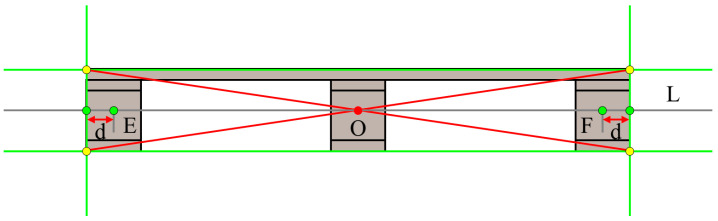
Schematic diagram of keypoint extraction.

The ground where the forklift operates is flat, and pallets are placed flat on the flatbed truck. It is reasonable to assume that the pallet surface is parallel to the camera surface. In fact, the positioning of a pallet requires the determination of four degrees of freedom (DOFs), including 3D spatial coordinates and rotation angle. The pallets used in this paper have a common feature, that is, the front surface is planar and presents a regular rectangular shape, with four sides approximated as straight lines. The upper and lower edge contours of the pallet are longer and approximately horizontal, with an angle close to 0°, while the left and right edge contours are shorter and approximately vertical, with an angle close to 90°. We have designed a method to accurately extract keypoints on the front surface of the pallet from the 2D image that are used to calculate the pallet position and rotation angle. The geometric center of the pallet surface and the correction points of the boundary center points on both sides are used as keypoints, as shown in Figure 6.

**Figure 7:**
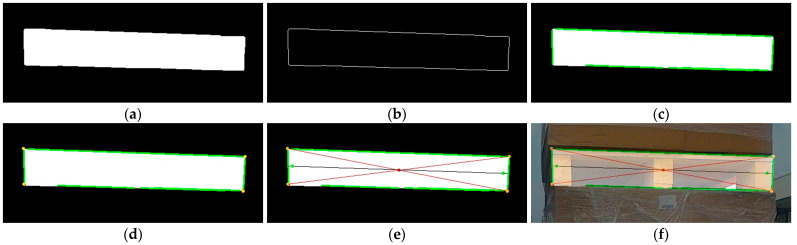
Graphic presentation of primary steps of keypoint extraction based on RGB image. (**a**) Segmentation image; (**b**) contour detection; (**c**) line extraction; (**d**) intersection clustering; (**e**) keypoint extraction; (**f**) overlay image.

**Figure 8:**
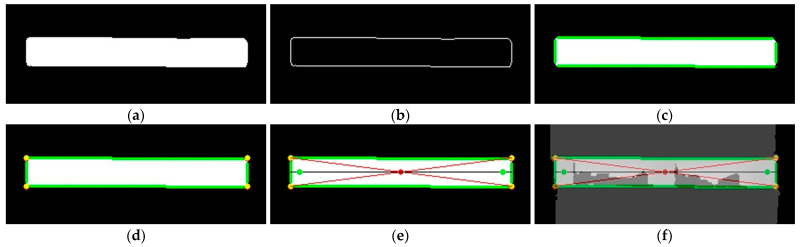
Graphic presentation of primary steps of keypoint extraction based on depth image. (**a**) Segmentation image; (**b**) contour detection; (**c**) line extraction; (**d**) intersection clustering; (**e**) keypoint extraction; (**f**) overlay image.

We take a single pallet as an example to illustrate the keypoint extraction process. First, the contour detection method is performed to extract the pallet contour from the image segmentation result, followed by the extraction of the boundary straight lines using PPHT. The lines close to 0° and 90° are screened based on the horizontal angle threshold range $\left[T_{1},\;T_{2}\right]$ and the vertical angle threshold range $\left[T_{3},\;T_{4}\right]$, respectively. The intersections of these two types of lines are then calculated and denoted as the set P. Furthermore, the intersection points in set P are classified into four categories using the K-means clustering algorithm, with the cluster centers representing the endpoints of the pallet boundaries. The intersection point of the diagonals serves as the keypoint for calculating the pallet position and is denoted as O. According to the four endpoints of the pallet, we calculate the pixel coordinates of the centers of the left and right boundaries, respectively. However, the coordinate data near the pallet boundary are not stable, and the two center points may not actually be located on the front surface of the pallet, which may result in an inability to accurately obtain the 3D coordinates of the pallet. Therefore, a straight line is computed through these two center points, labeled as L. Subsequently, the corrected coordinate points are obtained by moving the two center points along this straight line towards the inside of the pallet by a distance of d pixels. These two corrected points are used as keypoints for calculating the rotation angle of the pallet, labeled as E and F, respectively. The primary steps of keypoint extraction are shown in Figure 7 and Figure 8. 

**Figure 9:**
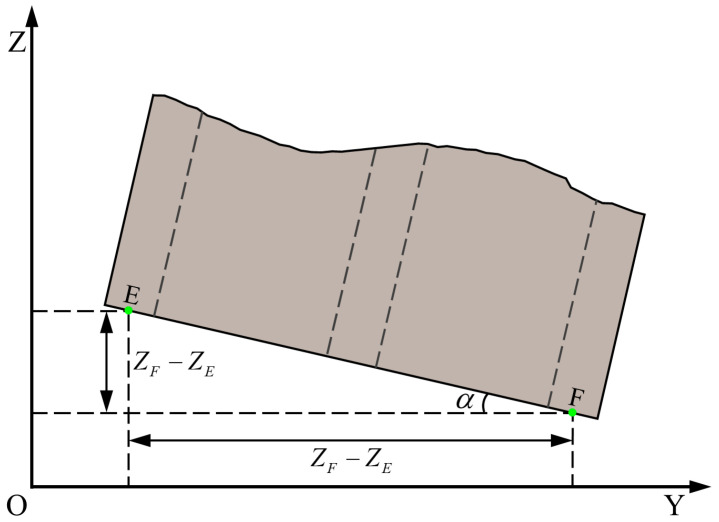
Schematic diagram of pallet rotation angle calculation.

The alignment of the RGB image and the depth image is achieved through the camera’s intrinsic and extrinsic parameters, and the depth image is mapped to the point cloud data. This process is automatically completed inside the camera. The 3D coordinates of the keypoints under the camera coordinate system are determined by indexing the pixel coordinates in the point cloud data. After eliminating invalid values such as infinite values and null values, the average value of multiple valid measurements is taken, and the 3D coordinates of the keypoints are recorded as $O(X_{C},Y_{C},Z_{C})$, $E(X_{E},Y_{E},Z_{E})$, and $F(X_{F},Y_{F},Z_{F})$, respectively. A schematic diagram of rotation angle calculation is shown in Figure 9. The rotation angle of the pallet is calculated as shown in Formula (1).

(1) $$\alpha =arctan\left(\frac{Z_{F}-Z_{E}}{X_{F}-X_{E}}\right)/pi\times 180{}^{\circ}$$

In Formula (1), α represents the rotation angle. $Z_{F}-Z_{E}$ represents the coordinate difference between E and F along the *Z*-axis direction. $X_{F}-X_{E}$ represents the coordinate difference between E and F along the *X*-axis direction.

### 2.4. Target Pallet Identification

In unloading scenarios, complex situations where pallets are stacked and closely arranged occur frequently. The unmanned forklift must be able to accurately search for the target pallet from multiple pallets presented in the camera’s field of view. To solve this problem, this paper proposes a method for searching for a target pallet. This method constrains the pixel coordinates and depth coordinates of the center points of all pallets and sets the priority of the pallets according to the unloading direction to locate the target pallet among many pallets.

**Figure 10:**
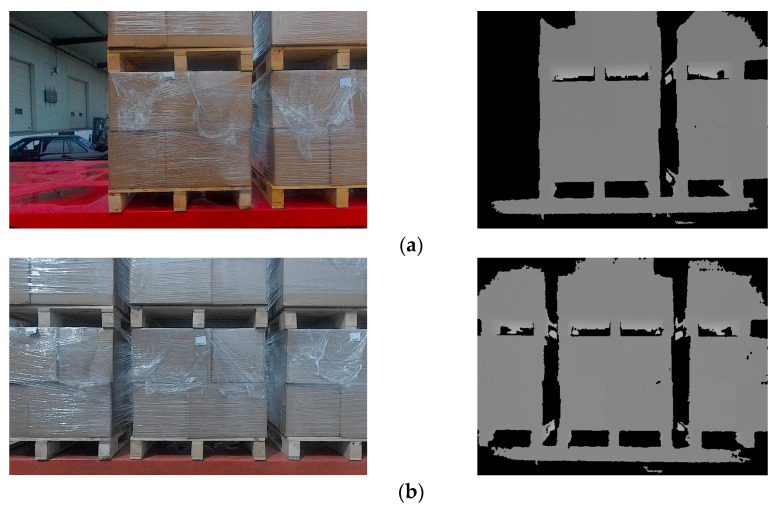

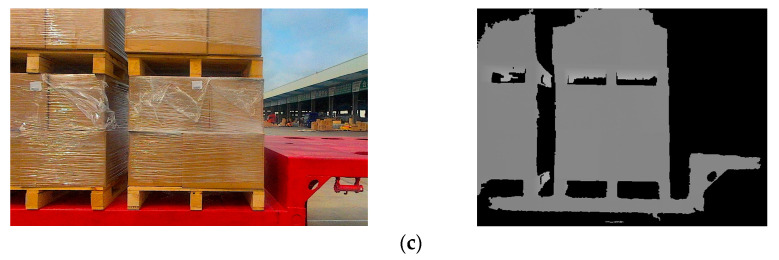
Multiple pallets appear in the camera’s field of view due to the close arrangement of goods. (**a**) Two columns of pallets are displayed, with only the column in the middle being complete. The column on the right side is incomplete and only partially visible, with no pallets on the left side. (**b**) Three columns of pallets are displayed, with only the column in the middle being complete. The columns on both the left and right sides are incomplete and only partially visible. (**c**) Two columns of pallets are displayed, with only the middle column being complete. The column on the right is incomplete and only partially visible, with no pallets on the right side.

**Figure 11:**
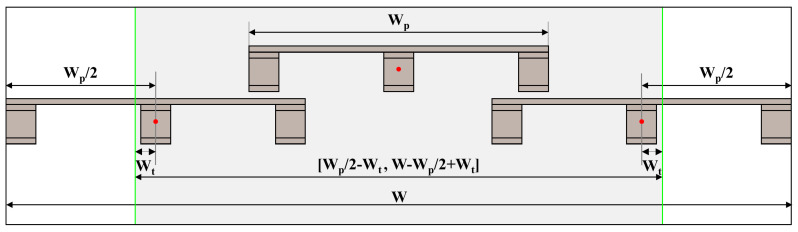
The width pixel coordinate constraint.

Since goods are usually closely arranged, multiple pallets may be present in the camera’s field of view at the same time, as shown in Figure 10. The width pixels occupied by the complete pallet that appears in the camera’s field of view are counted and denoted as $W_{P}$. Considering that the width pixel value occupied by the tilted pallet is often smaller than $W_{P}$, we introduce the width pixel adjustment amount $W_{T}$ to extend the range of the width pixel coordinate constraint, denoted as $\left[W_{P}/2-W_{T},\;{W-W}_{P}/2+W_{T}\right]$, as shown in Figure 11. Only pallets whose center point’s width pixel coordinates are within this range are considered as potential target pallets.

**Figure 12:**
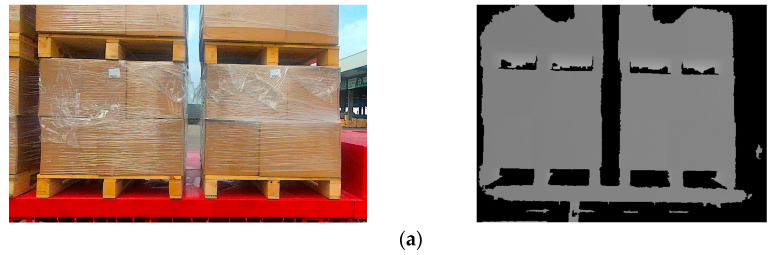

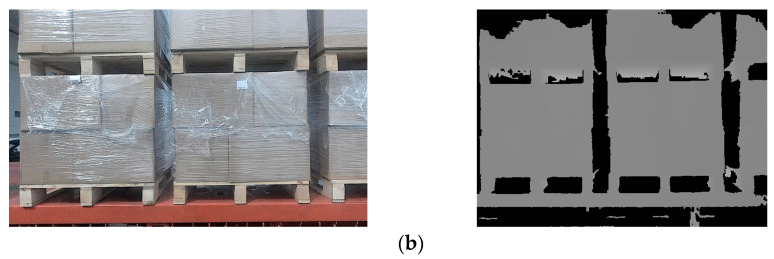
More complete pallets appear in the camera’s field of view due to deviation between the unmanned forklift’s observation position and the pallet position. (**a**) When unloading from the front of the flatbed truck, two complete columns of pallets are displayed in the camera’s FOV; (**b**) when unloading from the rear of the flatbed truck, two complete columns of pallets are displayed in the camera’s FOV.

**Figure 13:**
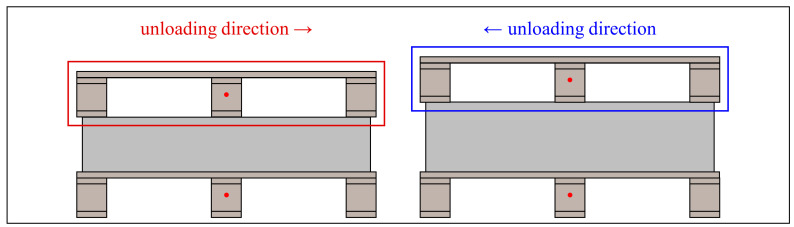
Unloading direction constraint.

When there is a significant deviation between the unmanned forklift’s observation position and the pallet position, a greater number of complete pallets can be observed in the camera’s field of view, as shown in Figure 12. The center points of these pallets all meet the width pixel coordinate constraints. The priority of pallets is set according to the unloading direction to ensure that the forklift promptly handles the pallets at the highest priority position, as shown in Figure 13. When an unmanned forklift unloads goods from the rear of a flatbed truck, pallets on the left side of the camera’s field of view are given the highest priority. Conversely, when the unmanned forklift unloads goods from the front of the flatbed truck, those on the right side are prioritized. The opposite rule applies to pallets on the other side of the flatbed truck.

**Figure 14:**
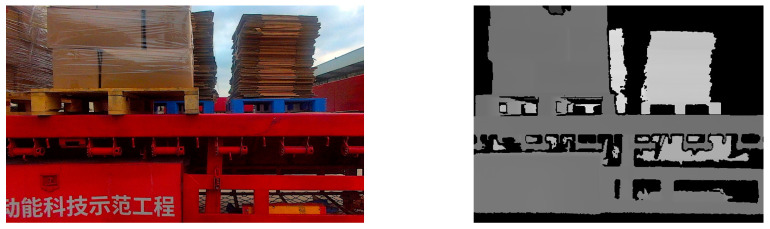
Pallet on the opposite side appears in the camera’s field of view.

**Figure 15:**
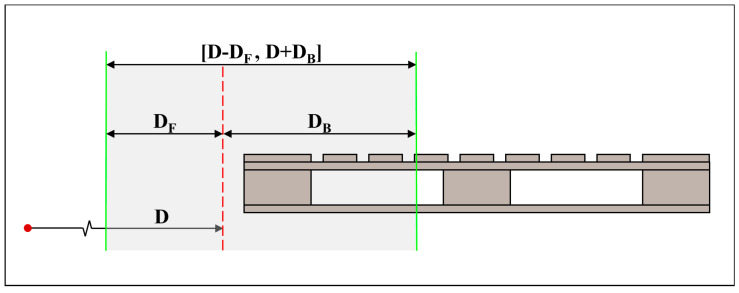
The depth distance coordinate constraint.

Unloading is carried out simultaneously by two unmanned forklifts from both sides of the flatbed truck, so the pallet on the opposite side may appear in the camera’s field of view, as shown in Figure 14. To minimize the impact of the rear pallet, with the forklift observation distance D as a reference, we introduce the depth distance adjustment amount $D_{F}$ and $D_{B}$ to expand the range of the depth distance coordinate constraint, denoted as $\left[D-D_{F},\;D+D_{B}\right]$, as shown in Figure 15. Only pallets whose center point’s depth coordinates are within this range are considered as potential target pallets. 

**Figure 16:**
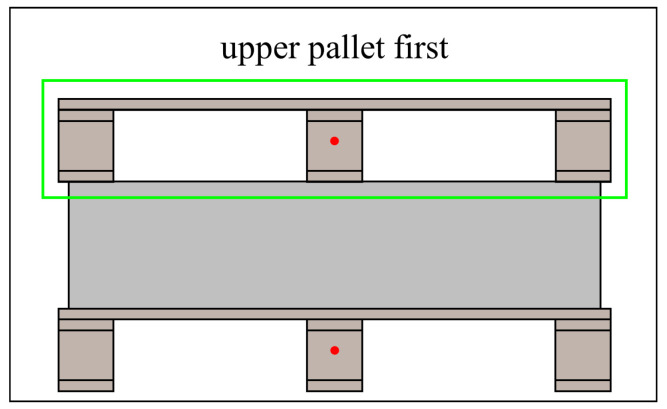
The height pixel coordinate constraint.

For pallets stacked in multiple layers on the same side, the upper pallet has higher priority, and the lower pallet has lower priority, as shown in Figure 16. Therefore, the pallet on top is selected as the target pallet.

**Figure 17:**
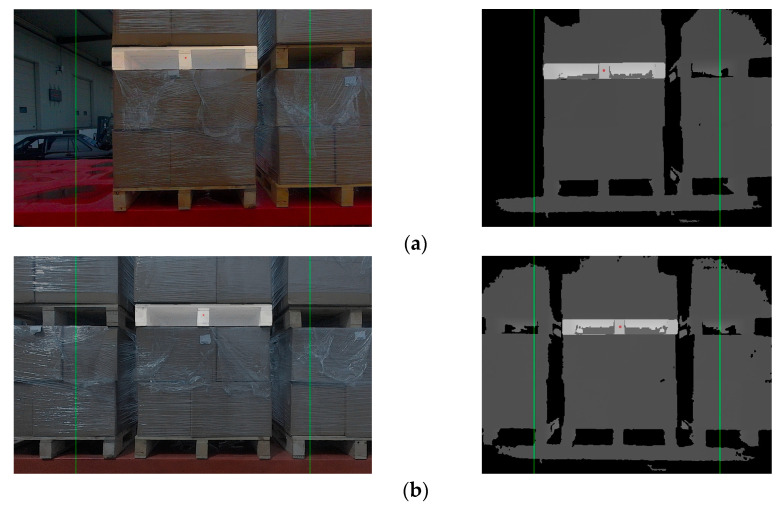

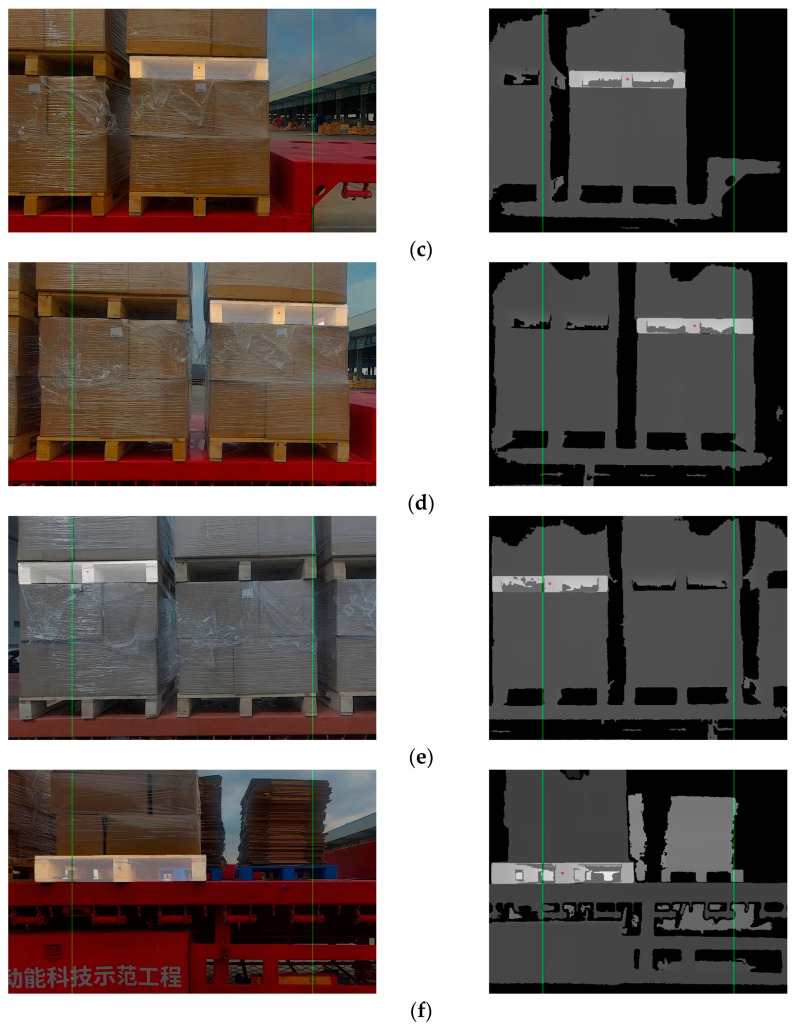
Positioning results of the target pallet in complex pallet arrangements. (**a**) The image is derived from Figure 10a, with the target pallet being the topmost complete pallet in the middle column. (**b**) The image is derived from Figure 10b, with the target pallet being the topmost complete pallet in the middle column. (**c**) The image is derived from Figure 10c, with the target pallet being the topmost complete pallet in the middle column. (**d**) The image is derived from Figure 12a. When unloading from the front of the flatbed truck, the target pallet is the topmost complete pallet in the rightmost column. (**e**) The image is derived from Figure 12b. When unloading from the rear of the flatbed truck, the target pallet is the topmost complete pallet in the leftmost column. (**f**) The image is derived from Figure 14, with the target pallet being the one located on the current side.

By setting constraints and priorities reasonably, unmanned forklifts can accurately find the target pallet in complex pallet arrangements. The positioning results of the target pallets are shown in Figure 17.

### 2.5. BDS Data Acquisition

**Figure 18:**
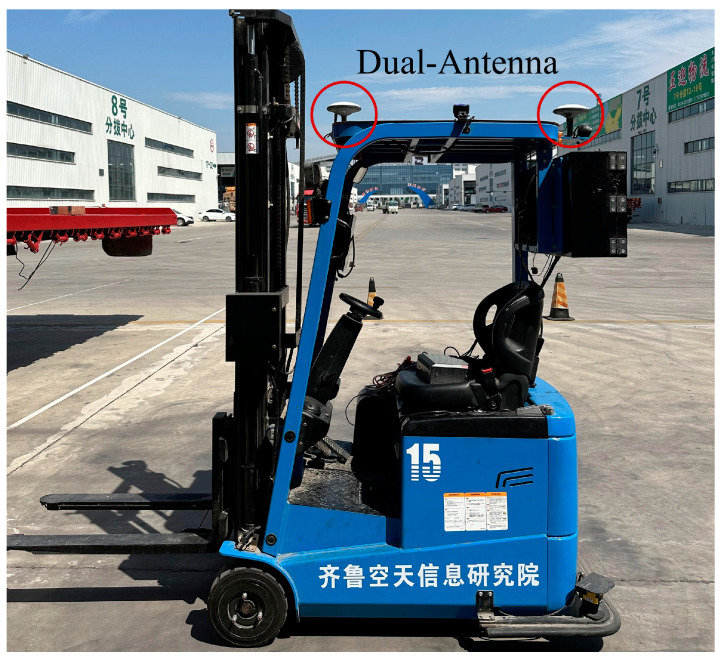
Dual-antenna installation position diagram.

A BDS reference station has been constructed in the business logistics park to provide high-precision positioning services for vehicle positioning devices. BDS-integrated inertial navigation outputs the position and yaw angle of the unmanned forklift at a high frequency. The dual-antenna BDS mobile receiver is installed on the roof along the longitudinal axis of the forklift as shown in Figure 18. The unmanned forklift relies on dual antennas to receive signals, so the forklift position coordinates actually refer to the longitude, latitude, and altitude coordinates of the main antenna in the China Geodetic Coordinate System 2000 (CGCS2000), denoted as (B,L, H). The yaw angle is the angle between the longitudinal axis of the forklift and the true north direction, ranging from 0° to 360°, denoted as θ. In order to facilitate subsequent calculation and processing, we convert the yaw angle of the forklift to the angle between the longitudinal axis of the forklift and the true east direction and adjust the angle range between −180° and 180°.

### 2.6. Coordinate Transformation

**Figure 19:**
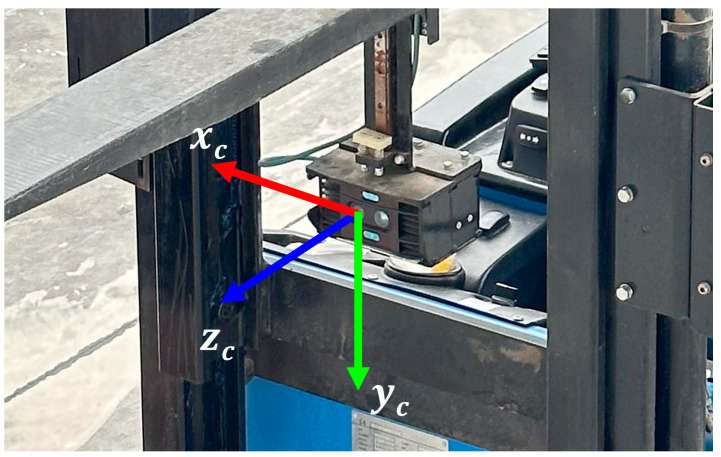
The camera coordinate system.

To achieve the localization and navigation of the forklift, it is necessary to transform the camera coordinate system into the forklift navigation coordinate system. The camera coordinate system is defined in Figure 19. Since the forklift does not use height information for navigation, the height information is ignored, and the camera coordinate system is dimensionally reduced. Then, the coordinates of the target pallet in the camera plane coordinate system are simplified to $\left(X_{C},Z_{C}\right)$. 

The forklift navigation coordinate system is established as a plane rectangular coordinate system, with its *Y*-axis representing the true north direction and its *X*-axis representing the true east direction. The coordinates of the main antenna in the forklift navigation coordinate system $\left(X_{B},Y_{B}\right)$ can be obtained by transforming the coordinates of the main antenna in the CGCS2000 coordinate system (B,L, H). First, we transform the BeiDou positioning coordinates (B,L, H) to the Earth-Centered, Earth-Fixed (ECEF) coordinates $\left(X_{b},Y_{b},Z_{b}\right)$. The transformation methods are shown in Formulas (2)–(4) [27,28].

(2) $$X_{b}=(N+H)cos\;B\cdot cos\;L$$

(3) $$Y_{b}=(N+H)cos\;B\cdot sin\;L$$

(4) $$Z_{b}=\left[(1-e^{2})N+H\right]sin\;B$$

where N is the prime vertical radius of curvature and e is the first eccentricity of the Earth. The respective calculation methods are shown in Formulas (5) and (6).

(5) $$N=\frac{a}{\sqrt{1-e^{2}{sin}^{2}\;B}}$$

(6) $$e=\sqrt{2f-f^{2}}$$

Here, a is the Earth’s semi-major axis with a value of 6,378,137 m, and f is the Earth’s flattening with a value of 1/298.257222101. 

Next, we transform the ECEF coordinates to the forklift navigation coordinate system. The origin of the forklift navigation coordinate system, which is the known reference point, has coordinates $\left(B_{O},L_{O},H_{O}\right)$ in the OGCS2000 coordinate system and $\left(X_{O},Y_{O},Z_{O}\right)$ in the ECEF coordinate system. The transformation methods are shown in Formulas (7) and (8) [28].

(7) $$X_{B}=-sin\;L_{O}\left(X_{b}-X_{O}\right)+cos\;L_{O}\left(Y_{b}-Y_{O}\right)$$

(8) $$Y_{B}=-sin\;B_{O}\cdot cos\;L_{O}\left(X_{b}-X_{O}\right)-sin\;B_{O}\cdot sin\;L_{O}\left(Y_{b}-Y_{O}\right)+cos\;B_{O}\left(Z_{b}-Z_{O}\right)$$

**Figure 20:**
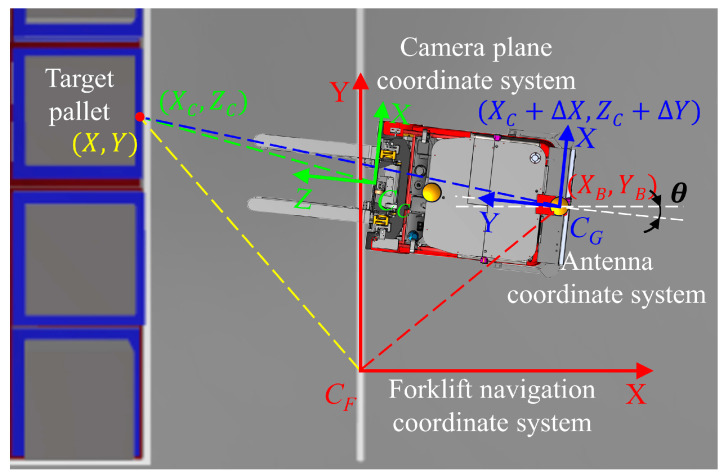
The spatial relationship among the camera plane coordinate system, the antenna coordinate system, and the forklift navigation coordinate system.

For clarity, we denote the camera plane coordinate system as $C_{C}$, the main antenna coordinate system as $C_{G}$, and the forklift navigation coordinate system as $C_{F}$. The spatial relationships among the three are illustrated in Figure 20. 

Assuming that the origin of the camera plane coordinate system moves by ∆X along the *X*-axis direction and ∆Y along the *Y*-axis direction until it coincides with the antenna coordinate system, the coordinates of the target pallet in the antenna coordinate system are $\left(X_{C}+∆X,Z_{C}+∆Y\right)$. Furthermore, the forklift navigation coordinate system can be obtained from the antenna coordinate system through rotation and translation. The coordinates of the target pallet in the forklift navigation coordinate system can be calculated using the following formulas:

(9) $$X=X_{B}-\left(Z_{C}+∆Y\right)cos\;\theta +(X_{C}+∆X)sin\;\theta$$

(10) $$Y=Y_{B}+\left(Z_{C}+∆Y\right)sin\;\theta +(X_{C}+∆X)cos\;\theta$$

## 3. Experiments

### 3.1. Forklift and Sensor Selection

The hardware used in this research included a forklift body, an RGB-D camera, and a dual-antenna BDS mobile receiver. The forklift body (model: TKA1535F905) was produced by Hefei Banyitong Science and Technology Developing Co., Ltd., located in Hefei, China, with a motion control precision of 3 cm. The RGB-D camera (model: TL460-S1-E1) was manufactured by Shanghai Tuyang Information Technology Co., Ltd., in Shanghai, China, with a range error of 9 mm at a distance of 2 m. The dual-antenna BDS mobile receiver (model: X1–5) was produced by Hunan Bynav Technology Co., Ltd., based in Changsha, China. This receiver can achieve a Real-Time Kinematic (RTK) positioning accuracy of 1.2 cm. Specifically, the system maintains its high precision when there are no obstacles within 15 degrees of the circumferential height angle above the receiver. During the loading process, ensuring that there are no obstructions above the forklift’s path is essential for the BDS receiver to reliably acquire satellite signals and achieve the stated positioning accuracy.

### 3.2. Experimental Site

**Figure 21:**
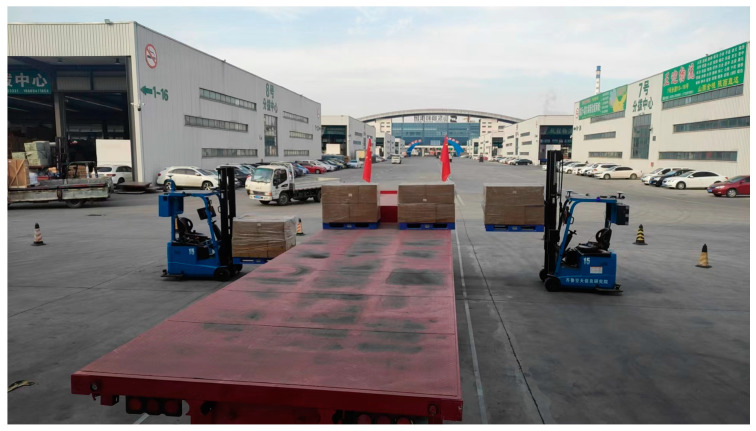
Map of the experimental field scene.

The evaluation experiments of this method were conducted at Shunhe International Intelligent Logistics Park in Linyi City, Shandong Province, as shown in Figure 21. The flatbed truck was parked along the stop line and multiple pallets were placed on the flatbed truck. The unmanned forklift arrived at the observation position on the side of the flatbed truck to conduct pallet localization measurement and estimate rotation angle. The experiment completed the positioning measurement of pallets at three different positions. For each observation position, the position coordinates and rotation angle data of the pallet were recorded 20 times consecutively.

### 3.3. Pallet Image Segmentation Dataset

The dataset for pallet image segmentation consists of 1078 RGB images and 992 depth images. Eighty percent of the data was allocated to the training set, while the remaining twenty percent was used for validation, without establishing a separate test dataset. For RGB images with a resolution of 1184 × 720, the batch size was set to 32, while for depth images with a resolution of 640 × 480, the batch size was set to 16. The initial learning rate was set to 0.0003 and was dynamically adjusted using the cosine annealing function over 200 epochs. These parameter settings were chosen to balance training stability and convergence speed, ensuring effective segmentation performance for both RGB and depth images.

### 3.4. Parameter Setting

#### 3.4.1. Preprocessing Parameter Selection

Considering the influence of factors such as the RGB-D camera’s field of view, measurement accuracy, and fork arm length, the vertical distance D between the observation position and the stop line was set to 2 m to ensure that the camera could acquire a complete image of the pallet. The depth threshold $D_{T}$ was set to 3 m to reduce interference of non-target-object pixels in the depth image by eliminating depth data greater than 3 m. A Gaussian filter was applied to smooth the image, with the window size set to 9 × 9.

#### 3.4.2. Keypoint Extraction Parameter Settings

Taking into account that the pallet may not be strictly placed horizontally, the angle threshold range can be appropriately expanded to allow for a certain degree of tilt in the pallet. The horizontal angle threshold range $\left[T_{1},T_{2}\right]$ and vertical angle threshold range $\left[T_{3},T_{4}\right]$ were set to [−3°, 3°] and [85°, 95°], respectively. Horizontal line segments longer than 120 pixels and vertical line segments longer than 20 pixels were extracted from the RGB image. Horizontal line segments longer than 50 pixels and vertical line segments longer than 10 pixels were extracted from the depth image. According to the size of the pixels occupied by the pallet leg, the pixel distance moved by the midpoint of the short edge was appropriately adjusted to ensure that the corrected coordinate points were located on the pallet surface. In this experiment, d was set to 10 pixels.

#### 3.4.3. Target Pallet Constraint Threshold Settings

The width pixels $W_{P}$ occupied by the pallet in the RGB image and depth image were approximately 460 pixels and 260 pixels, respectively. The width pixel adjustment amounts $W_{T}$ were set to 25 pixels and 20 pixels, so the width pixel coordinate constraints were [205, 979] and [115, 525], respectively. The depth distance adjustment amount $D_{F}$ and $D_{B}$ were set to 0.3 m and 0.5 m, respectively, so the depth distance coordinate constraint was [1.7, 2.5].

## 4. Evaluation

The absolute error (AE) is a commonly used error evaluation index and represents the absolute difference between the measured value and the reference value, as illustrated in Formula (11). ${AE}_{i}$ was calculated separately for multiple measurement data obtained at each observation position, and the maximum value ${AE}_{max}$, the minimum value ${AE}_{min}$, and the mean value MAE were further calculated, as shown in Formulas (12), (13), and (14), respectively.

(11) $${AE}_{i}=\left|r_{i}-R\right|$$

(12) $${AE}_{max}=max\left({AE}_{1},{AE}_{2},\ldots {,AE}_{n}\right)$$

(13) $${AE}_{min}=min\left({AE}_{1},{AE}_{2},\ldots {,AE}_{n}\right)$$

(14) $$MAE=\frac{1}{n}\sum_{i=1}^{n}\left({AE}_{1},{AE}_{2},\ldots {,AE}_{n}\right)$$

For an assessment of the dispersion of the observed data, the standard deviation (STD), presented in Formula (15), serves as an evaluation metric for quantifying the spread of measured values.

(15) $$STD=\sqrt{\frac{1}{n}\sum_{i=1}^{n}{\left(r_{i}-\bar{r}\right)}^{2}}$$

In the above formulas, R represents the reference value, $r_{i}$ represents the measurement value for the *i*-th time, n represents the number of measurements at the same observation position, and $\bar{r}$ represents the average value of the n measurements.

The results of target pallet segmentation and keypoint extraction based on RGB images and depth images are shown in Table 3 and Table 4, respectively.

The evaluation indices were calculated from the four degrees of freedom: X, Y, Z coordinates and rotation angle. The results are shown in Table 5 and Table 6.

Judging from the results, the AE of the localization results based on RGB images in the X, Y, and Z directions is less than 18 mm, with an STD of less than 2.2 mm. The AE of the rotation angles is less than 0.34°, with an STD of less than 0.20°, which indicates that the prediction results have a low degree of dispersion. In comparison, the localization results based on depth images show AE of less than 20 mm in the X, Y, and Z directions, with an STD of less than 2.1 mm. The AE of the rotation angles is less than 0.37°, with an STD of less than 0.22°, also demonstrating a low degree of dispersion. However, the results based on depth images exhibit a slight decrease in pallet localization accuracy. This difference can be attributed to the different resolutions of the two types of images, as well as variations in texture and color information. Specifically, the resolution of the depth images is 640 × 480, whereas the resolution of the RGB images is 1184 × 720. The lower resolution of the depth images results in less detailed information. Additionally, RGB images provide richer texture and color information, while depth images lack these detailed data, thereby affecting the accuracy of pallet detection and localization. Nevertheless, both methods provide accurate estimates for the rotation angles, which is related to their use of relative distances in calculations. The automatic unloading test conducted at the logistics site demonstrated that the accuracy meets the requirements.

## 5. Conclusions

This study aims to address the challenges of locating target pallets in outdoor unmanned forklift unloading scenarios, particularly due to uncontrollable lighting conditions and the close stacking of pallets. We propose a method for high-precision positioning and rotation angle estimation for a target pallet, utilizing the BDS and vision. This method has been successfully implemented in real unloading scenarios. The experimental results indicate that the pallet positioning error is less than 20 mm, and the rotation angle error is within 0.37°, thus meeting the accuracy requirements for automated forklift operations. Looking ahead, we plan to integrate depth and color channels to enhance pallet recognition. This approach aims to leverage the rich texture and color information available in RGB images and the strong resistance to lighting variations offered by depth images, ultimately improving both the accuracy and stability of pallet positioning. In particular, this integration seeks to address the specific challenge of recognizing pallets when two pallets are placed closely together with nearly aligned front faces. Additionally, some parameters related to keypoint extraction and target pallet constraints are still manually adjusted. Therefore, we intend to design and implement adaptive adjustment mechanisms for these parameters.

## 6. Patents

This paper has resulted in a patent with application number CN202310770170.7, titled “Method and Device for Segmentation and Positioning of Forklift Pallets, and Intelligent Forklift”.

## Figures and Tables

**Table 1:** The architecture of DDRNet-23-slim.

Stage	Output	DDRNet-23-Slim	
conv1	112 × 112	3 × 3, 32, stride 2	
conv2	56 × 56	3 × 3, 32, stride 2	
		$\left[\begin{array}{c} 3\times 3,32\\ 3\times 3,32 \end{array}\right]\times 2$	
conv3	28 × 28	$\left[\begin{array}{c} 3\times 3,64\\ 3\times 3,64 \end{array}\right]\times 2$	
conv4	14 × 14, 28 × 28	$\left[\begin{array}{c} 3\times 3,128\\ 3\times 3,128 \end{array}\right]\times 2$	$\left[\begin{array}{c} 3\times 3,64\\ 3\times 3,64 \end{array}\right]\times 2$
		Bilateral fusion	
conv5_1	7 × 7, 28 × 28	$\left[\begin{array}{c} 3\times 3,256\\ 3\times 3,256 \end{array}\right]\times 2$	$\left[\begin{array}{c} 3\times 3,64\\ 3\times 3,64 \end{array}\right]\times 2$
		Bilateral fusion	
		$\left[\begin{array}{c} 1\times 1,256\\ 3\times 3,256\\ 1\times 1,512 \end{array}\right]\times 1$	$\left[\begin{array}{c} 1\times 1,64\\ 3\times 3,64\\ 1\times 1,128 \end{array}\right]\times 1$
conv5_2	7 × 7	High-to-low fusion	
		1 × 1, 1024	
	1 × 1	7 × 7 global average pool	
		1000-d fc, softmax	

**Table 2:** Information about some pallet types in the dataset.

Number	Material	Color	Dimensions (W × L × H) mm	Diagram
1	Wood	Wooden	1000 × 1200 × 150	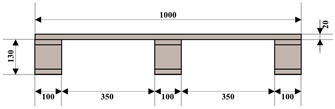
2	Plastic	Blue	1000 × 1200 × 150	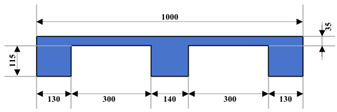

**Table 3:** Results of target pallet segmentation and keypoint extraction based on RGB images.

Serial Number	RGB Images	Image Segmentation Results	Keypoint ^1^ Recognition Results	Target Pallet Identification Results
1	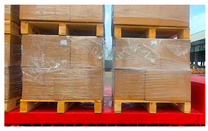	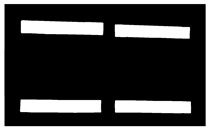	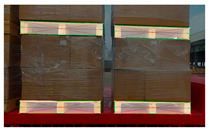	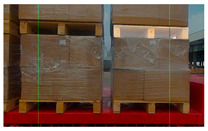
2	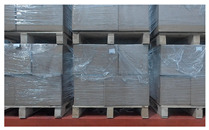	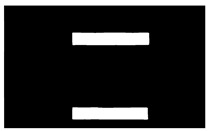	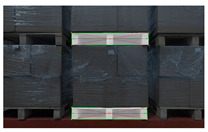	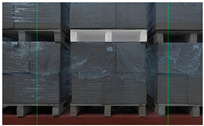
3	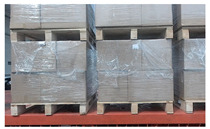	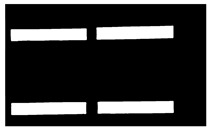	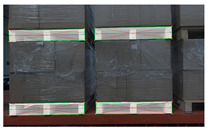	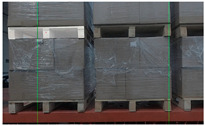

^1^ Keypoints are depicted as red and green circles in the images.

**Table 4:** Results of target pallet segmentation and keypoint extraction based on depth images.

Serial Number	Depth Images	Image Segmentation Results	Keypoint ^1^ Recognition Results	Target Pallet Identification Results
1	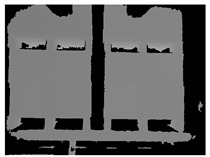	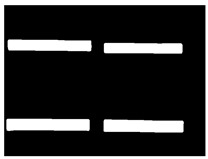	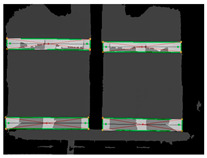	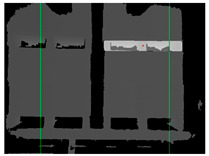
2	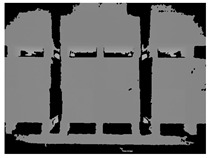	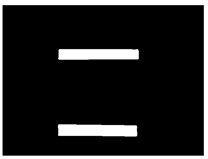	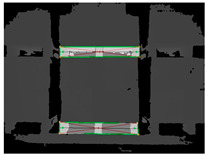	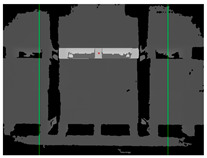
3	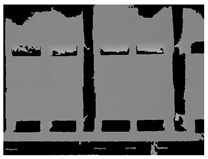	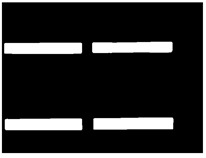	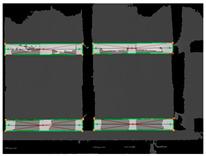	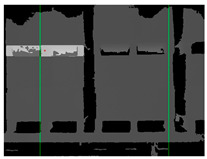

^1^ Keypoints are depicted as red and green circles in the images.

**Table 5:** Error between reference and predicted coordinates of target pallet from RGB images.

Serial Number	Degrees of Freedom	Error (mm/°)			
		${AE}_{min}$	${AE}_{max}$	MAE	STD
1	X	9.682	16.218	12.440	2.160
	Y	11.826	16.996	14.669	1.695
	Z	10.405	15.041	13.088	1.556
	Angle	0.012	0.318	0.182	0.200
2	X	8.172	14.183	10.964	2.004
	Y	10.537	16.543	13.198	1.771
	Z	10.313	15.679	13.061	1.750
	Angle	0.015	0.336	0.172	0.190
3	X	11.157	15.426	13.088	1.207
	Y	10.496	15.373	13.156	1.678
	Z	11.507	17.319	14.245	1.835
	Angle	0.016	0.313	0.180	0.198

**Table 6:** Error between reference and predicted coordinates of target pallet from depth images.

Serial Number	Degrees of Freedom	Error (mm/°)			
		${AE}_{min}$	${AE}_{max}$	MAE	STD
1	X	13.121	19.679	15.882	1.878
	Y	13.165	19.579	17.093	1.784
	Z	11.150	15.777	13.753	1.532
	Angle	0.018	0.349	0.185	0.211
2	X	10.091	15.830	13.665	1.671
	Y	12.549	19.914	16.645	1.815
	Z	10.993	16.960	14.085	2.033
	Angle	0.019	0.364	0.169	0.181
3	X	12.972	18.388	15.603	1.723
	Y	13.830	19.766	17.579	1.991
	Z	12.387	18.135	15.854	1.764
	Angle	0.007	0.358	0.177	0.206

## Data Availability

Embargo on data due to commercial restrictions.
